# 2844. Impact of Altered Mental Status on Antibiotic Prescribing in Patients with Pyuria

**DOI:** 10.1093/ofid/ofad500.2454

**Published:** 2023-11-27

**Authors:** Haley Boerckel, Lisa E Dumkow, Lacy Worden, Lisa Salvati, Andrew Jameson

**Affiliations:** Trinity Health Grand Rapids, Grand Rapids, Michigan; Trinity Health Grand Rapids, Grand Rapids, Michigan; Ascension Borgess, Kalamazoo, Michigan; Trinity Health Grand Rapids, Grand Rapids, Michigan; Trinity Health Grand Rapids, Grand Rapids, Michigan

## Abstract

**Background:**

The Infectious Diseases Society of America’s guidelines for the management of asymptomatic bacteriuria state that altered mental status (AMS) is non-specific and alone should not be classified as a symptom of urinary tract infection (UTI). Careful observation, rather than treatment, is recommended to avoid adverse medication events and antibiotic resistance. The purpose of this study was to evaluate the burden of antibiotic overtreatment, patient outcomes, and risk factors for treatment of AMS patients with asymptomatic pyuria within a community teaching hospital.

**Methods:**

This retrospective cohort study evaluated adult inpatients admitted with AMS and pyuria (10 WBC/hpf) between February 1, 2020 and October 1, 2021. The primary objective was to compare outcomes between patients with AMS treated for asymptomatic pyuria (AMS+Tx) vs. those without treatment (AMS-NoTx) using a composite endpoint of readmission for AMS or development of

Clostridiodes difficile within 30-days, or development of new antibiotic resistance within 90 days. Secondary outcomes included characterizing the burden of overtreatment and identifying risk factors for treatment of pyuria in patients with AMS. Multi variate logistic regression identified risk factors independently associated with treatment of asymptomatic pyuria in patients with AMS. Patients were excluded who had noted urinary symptoms, admission to critical care, history of renal transplant, urological surgery, co-infection, pregnancy, or neutropenia.

**Results:**

A total of 200 patients were included (AMS+Tx, n= 160; AMS-NoTx, n=40). No difference was observed between groups in the composite primary outcome (AMS+Tx 23.5% vs AMS-NoTx 18.1%, p=0.465). The burden of overtreatment was 80%. An alternative diagnosis of AMS occurred more frequently when antibiotics were withheld (AMS+Tx 65.6% vs.AMS-NoTx 87.5%, p=0.007). Urinalyses showing bacteria (OR 2.63; 95% CI 1.15-6.03) and positive urine culture (OR3.76; 95% CI 1.67-8.48) were associated with the use of antibiotics.

**Conclusion:**

Patient-related outcomes were similar between groups highlighting the burden of antibiotic overtreatment and need for stewardship interventions in inpatients with AMS and pyuria presenting without other urinary symptoms.

**Disclosures:**

**All Authors**: No reported disclosures

